# “Water? Which *Aedes aegypti* Pupa Needs It!”

**DOI:** 10.3390/insects17080770

**Published:** 2026-07-27

**Authors:** Muhammad Faizal Zulkifli, Jonathan Wee Kent Liew, Meng Li Wong, Lu Deng, Cheong-Huat Tan

**Affiliations:** Environmental Health Institute, National Environment Agency, 5008 Ang Mo Kio Ave 5, #06-13, Singapore 569874, Singapore; jonathan_liew@nea.gov.sg (J.W.K.L.); wong_meng_li@nea.gov.sg (M.L.W.); lu_deng@nea.gov.sg (L.D.); tan_cheong_huat@nea.gov.sg (C.-H.T.)

**Keywords:** *Aedes*, aquatic stage, mosquito pupae, emergence

## Abstract

Mosquito pupae are conventionally believed to require water for survival and subsequent emergence into adult mosquitoes. This study assessed emergence of *Ae. aegypti* mosquitoes from four substrates: (1) water; (2) moist filter paper; (3) moist filter paper with a wet cotton pad below it; and (4) double-meshed wire surface with a wet tissue below it. High levels of emergence (at least 90%) were obtained for all four substrates. These mosquitoes displayed comparable fitness levels as represented by their flight ability and mating success. The median survival curve of male mosquitoes showed no significant differences, but significant differences were observed between groups of female mosquitoes that emerged from different substrates. The results will be helpful for programmes that intend to improve the logistical handling of mosquitoes for field releases.

## Simple Summary

Our study showed that pupae can emerge on moist surfaces with no impact on fitness. We tested emergence from four different environments: (1) water, (2) moist paper, (3) moist paper with a wet cotton pad underneath, and (4) a double-meshed wire surface with a wet tissue underneath. The results showed that mosquitoes could emerge successfully in all conditions, with at least 90% survival rates. The mosquitoes that emerged from these conditions showed similar flying and mating abilities. However, survival rates varied among female mosquitoes depending on the substrate they emerged from.

## 1. Introduction

Mosquito larva undergoes four instar stages before moulting into the non-feeding, mobile pupae within which the adult mosquito tissues and structures develop. The pupal stage typically lasts about 2–3 days. When the pupa is fully developed, it stays on the water surface and the pupal skin splits on the thorax, allowing the adult mosquito to emerge [1].

While it has been conventionally believed that mosquito pupae can only survive and emerge in an upright position in water, our laboratory observed that *Aedes* pupae could complete development and emerge as adult on moist filter paper. A search revealed that older literature documented the emergence of adults from pupae when removed from water [2]. Adult *Aedes aegypti* can emerge from pupae on moist filter paper [3], or even from pupae dried at 24 h after pupation [4].

Through continued R&D, this biological attribute is now used in the large-scale release of millions of male *Wolbachia*-infected *Ae. aegypti* mosquitoes under Project *Wolbachia*-Singapore (*Wolbachia-Aedes* Mosquito Suppression Strategy). This finding can offer logistical and efficiency benefits, particularly for field sampling and mosquito release programmes [5,6]. It is especially important for the operation and success of mosquito release programmes involving adult releases and may also potentially be used in the design of pupae release stations.

The current study details the emergence of adult *Ae. aegypti* mosquitoes from pupae maintained on three different moist surfaces, and assesses their corresponding fitness in comparison to adults that emerged on water. The emergence of adult *Ae. albopictus*, *Culex quinquefasciatus* and *Anopheles epiroticus* mosquitoes from pupae on moist surfaces was also observed.

## 2. Materials and Methods

### 2.1. Rearing Conditions

*Wolbachia* (*w*AlbB)-infected *Aedes aegypti* (henceforth referred to as *Ae. aegypti*) were reared at a density of 1500 larvae in 3 L of reverse osmosis (RO) water and fed with Tetramin fish food (Tetra^®^ Werke GmbH, Melle, Germany). Rearing was conducted in an environmentally controlled room, with a temperature of 27.5 ± 1.0 °C, relative humidity of 75.0 ± 10.0% and photoperiod of 12:12 (L:D) hours. Male and female pupae were separated using the Fay–Morlan separator (WBK-P0001-V1, Wolbaki, Guangzhou, China) and collected on day 6 after egg hatching. Experiments for each substrate were run in triplicate s, and each experiment was repeated three times, unless stated otherwise.

### 2.2. Emergence of Ae. aegypti on Different Substrates

**Figure 1:**
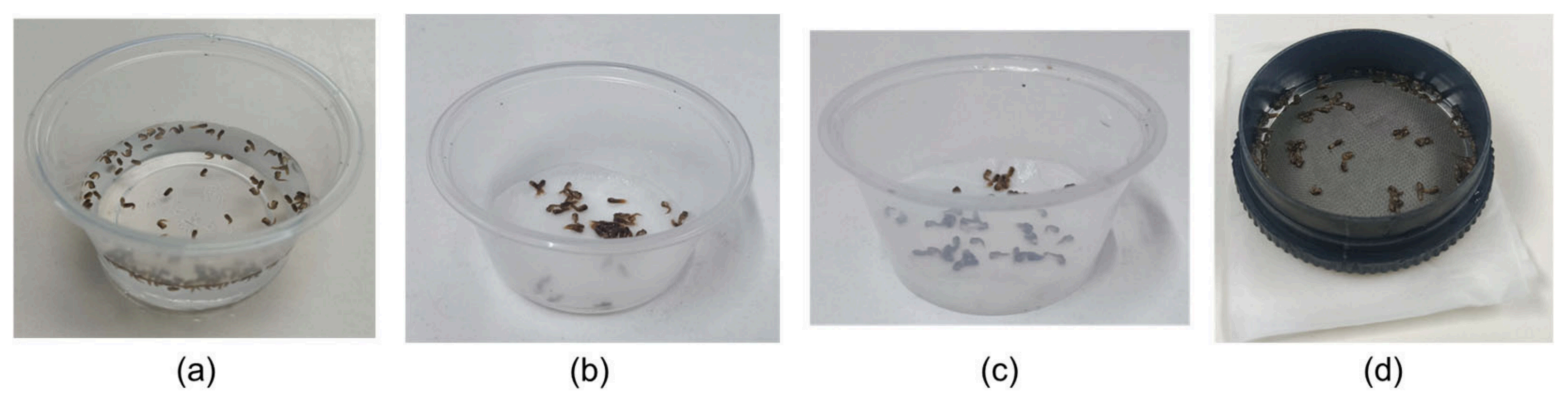
The four substrates in which the pupae were placed. (**a**) In water (control); (**b**) on a moist filter paper, where excess water was removed; (**c**) on a moist filter paper, with a wet cotton pad below it, and excess water removed; (**d**) on a double-meshed wire surface, with a wet tissue below it.

For each replicate, one hundred and fifty *Ae. aegypti* pupae of each gender were placed in four different substrates: (1) water (as a control); (2) on a moist filter paper (henceforth termed ‘moist’); (3) on a moist filter paper, with a wet cotton pad below it (henceforth termed ‘wet’); and (4) on a double-meshed wire surface (Orinno Technology Pte Ltd., Singapore), with a wet tissue below it (henceforth termed ‘double-meshed wire’) (Figure 1). The filter paper used was creped, qualitative, technical paper of grade 39/N (Sartorius, Goettingen, Germany) cut into circles with a diameter of 4 cm. The double-meshed wire surface, enclosed in a cap format (diameter = 4.8 cm), was able to provide moisture by holding a layer of water between them. The double-meshed cap with its container is currently used to hold the male pupae prior to emergence in Project *Wolbachia*-Singapore. The wet tissue slows down the evaporation of the layer of water. The pupae on the different substrates were allowed to emerge in individual 30 cm × 30 cm × 30 cm cages. The number of adult mosquitoes which emerged were counted hourly, where operationally possible, starting from 19 h after placement of pupae on these substrates to determine the emergence rate of mosquitoes over time. The number of dead pupae was also recorded.

### 2.3. Flight Ability of Male Ae. aegypti Mosquitoes That Emerged from Different Substrates

From each substrate group, about 80–120 male *Ae. aegypti* mosquitoes that emerged within a 7 h collection period (19–26 h after placement on substrates) were placed in 30 cm × 30 cm × 30 cm cages. Adult mosquitoes were fed with 10% sucrose solution in accordance with the standardised FAO/IAEA Flight Test protocol for quality control of sterile mosquitoes [7]. Mosquitoes aged five to six days post-emergence were used for the flight test. The Flight Test Device was used to assess the flight ability of adult male mosquitoes, which was determined by their escape rate. For two hours, male mosquitoes were attracted and stimulated to escape from the tube organ using the BG lure (Bioagents, AG, Regensburg, Germany). The number of mosquitoes that were found outside the tube organ (escaped mosquitoes) and inside the tube organ (non-escaped mosquitoes) were recorded, and the escape rate was defined as the percentage of escaped mosquitoes relative to the total number of mosquitoes at the beginning of the test.

### 2.4. Longevity of Male and Female Ae. aegypti Mosquitoes That Emerged from Different Substrates

From each substrate group, about 120–275 male or female *Ae. aegypti* mosquitoes that emerged within a 9 h collection period (19–28 h after placement on substrates) were placed separately in 30 cm × 30 cm × 30 cm cages. As female mosquitoes emerged later than male mosquitoes, the overall collection window was extended to 9 h to ensure a sufficient number of both sexes was collected. Adult mosquitoes were fed with 20% sucrose solution ad libitum. The longevity tests began three days later, when the sucrose solution was removed and replaced with water. Dead mosquitoes were recorded and removed daily, until all mosquitoes in the cages were dead. Kaplan–Meier log-rank analysis was performed and the survival curve recorded.

The longevity test performed in this manner simulates the availability of the resources that male adult *Ae. aegypti* mosquitoes may obtain before and after release in Project *Wolbachia*-Singapore: the male adult mosquitoes are provided with a 20% sucrose solution prior to release; upon their release into the environment, it is assumed that water is the most basic resource that they can obtain.

### 2.5. Mating Success of Ae. aegypti Mosquitoes That Emerged from Different Substrates

The insemination rate was assessed to observe the effect of the duration spent on the substrate prior to emergence on the mating success of the mosquitoes. From each substrate group, two groups of mosquitoes were sampled: (1) those that emerged within 19 h from placement on the substrates (henceforth termed ‘early’), and (2) those that emerged after 28 h since the pupae were placed on the substrate (henceforth termed ‘late’).

To assess the mating ability of male mosquitoes, one male mosquito was added to a paper cup (top diameter: 9 cm, bottom diameter: 7.2 cm, height: 10 cm) containing three virgin female mosquitoes (emerged in water). Correspondingly, to assess the mating ability of such female mosquitoes, one female mosquito was added to a cup containing one male mosquito (emerged in water). They were allowed to mate for three days, and 20% sucrose solution was provided throughout. At the end of three days, the female mosquitoes were dissected to check for insemination in the spermathecae.

Five replicates were prepared for each group of mosquitoes in their substrate group. These experiments were performed twice.

### 2.6. Emergence of Aedes albopictus, Culex quinquefasciatus and Anopheles epiroticus on Moist Surfaces

To observe whether other mosquito species could also emerge on moist surfaces, fifty pupae (a mix of male and female) of each laboratory-reared species, namely *Aedes albopictus*, *Culex quinquefasciatus* and *Anopheles epiroticus*, were placed in water, on a moist filter paper and on a double-meshed wire surface (Figure 1a,b,d), respectively. Based on the findings from *Ae. aegypti* experiment, *Ae. albopictus*, *Cx. quinquefasciatus* and *An. epiroticus* were not evaluated on the “wet” substrate as it did not provide any apparent advantage over the moist filter paper. Subsequent experiments therefore focused on comparing the water control with the moist filter paper and double-meshed wire substrates. The number of adult mosquitoes which emerged were counted at 24 h, 40–48 h and >60 h after the placement of pupae on these substrates. The number of dead pupae was also determined. 

### 2.7. Data Analysis

Data were primarily recorded and analysed in Microsoft Excel. Unless stated otherwise, further statistical analyses were performed using R language (4.4.0) in the RStudio environment (version 2024.04.0). A non-parametric Kruskal–Wallis test, with an alpha level of 0.05, was performed using the function kruskal.test(). Post hoc comparisons were performed using Dunn’s test using the function dunn.test() in the dunn.test package https://doi.org/10.32614/CRAN.package.dunn.test (accessed on 30 June 2026), and the *p*-values were corrected using the Bonferroni method.

Kaplan–Meier survival analysis was carried out using the survminer https://doi.org/10.32614/CRAN.package.survminer (accessed on 30 June 2026) and survival https://doi.org/10.32614/CRAN.package.survival (accessed on 30 June 2026) packages, to analyse the longevity of the mosquitoes. Pair-wise log-rank comparisons of the survival curves were subsequently conducted. With a Bonferroni correction, statistical significance was accepted at *p* < 0.008. Statistical significance for the differences in the emergence rates of mosquitoes placed in different substrates was assessed using the CGGC permutation test http://bioinf.wehi.edu.au/software/compareCurves (accessed on 8 May 2024). The initial *p*-values were adjusted for multiple testing using Holm’s method [8].

Insemination success was analysed separately for the male and female mating assays using binomial generalised linear models with a logit link function. Analyses were conducted in R using the glm() function. For the male assay, the response was recorded as the number of females inseminated out of three. However, for the female assay, the response was recorded as either inseminated or not inseminated. For each assay, the age group, substrate condition, and their interaction were included as explanatory variables, with the experimental round included as a fixed effect. Likelihood ratio tests were performed with ANOVA, applied using the Chi-Square statistics to assess the age group × substrate interaction, overall substrate effect, and overall age group effect by comparing nested models. Where a significant interaction was detected, substrate effects were further examined separately within early and late groups. Statistical significance was assessed at α = 0.05.

## 3. Results

### 3.1. Impact of Substrates on the Emergence of Ae. aegypti Mosquitoes

The number of pupae on each substrate ranged from 148 to 150 per replicate, except on one occasion where 144 male pupae were placed on the moist substrate. *Aedes aegypti* emergence rates exceeded 94% across all substrates (Table 1, Supplementary Video S1). Among these, water yielded the highest emergence rates for both females (99.56%) and males (99.63%), which were significantly higher than for all other substrates. The sole exception was males emerging from the wet substrate, where emergence rates did not differ significantly from those for water.

**Figure 2:**
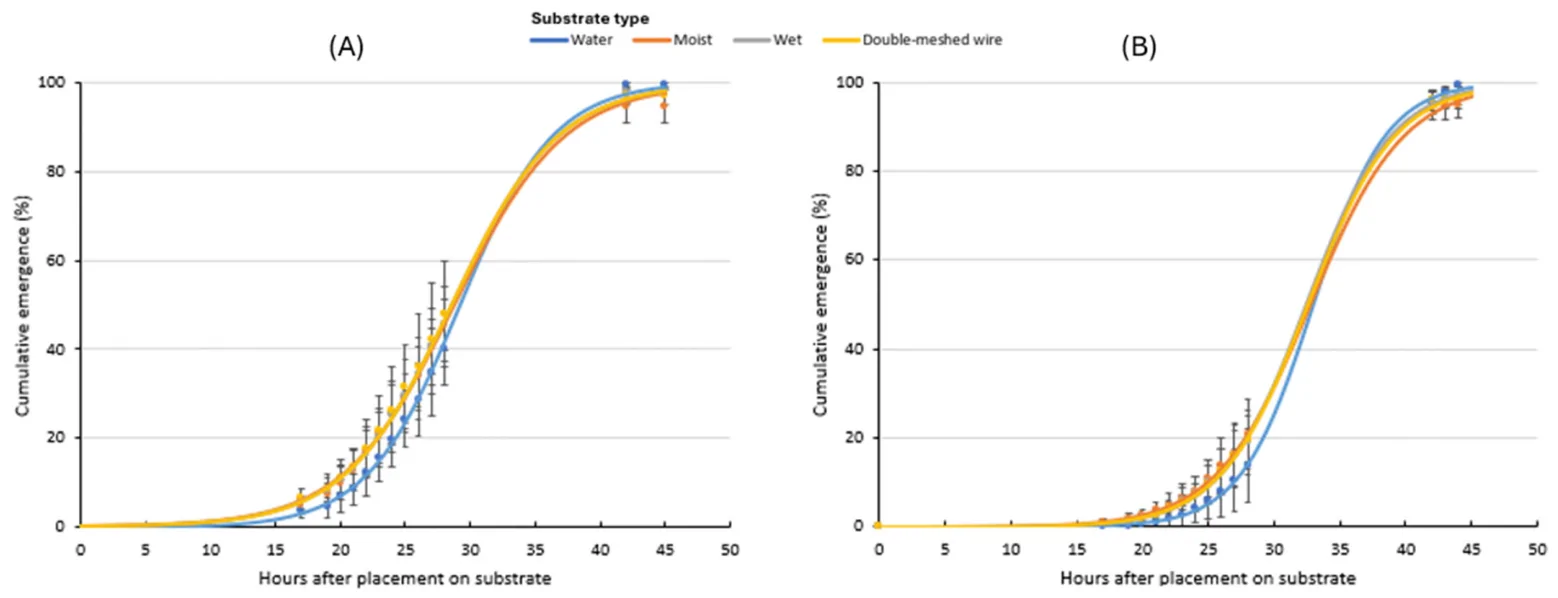
Emergence rates of mosquito species on different substrates: (**A**) male *Ae. aegypti* mosquitoes; (**B**) female *Ae. aegypti* mosquitoes. The error bars denote standard deviations.

As expected, the rate of *Ae. aegypti* male emergence was higher than that of females in the first 28 h across all substrates, but both converged to 95% by 42 h (Figure 2). At 24 h, an average of 19% to 26% of males had emerged, contrasting an average of 4% to 7% of females. Although the emergence rates of adults from pupae in water were lower, all pair-wise comparisons of emergence rates between the different substrates showed no statistical significance within each gender. Surface temperatures of the different substrates taken at relevant intervals were found to be similar (Figure S1).

### 3.2. Impact of Substrates on Flight Ability of Male Ae. aegypti Mosquitoes

**Figure 3:**
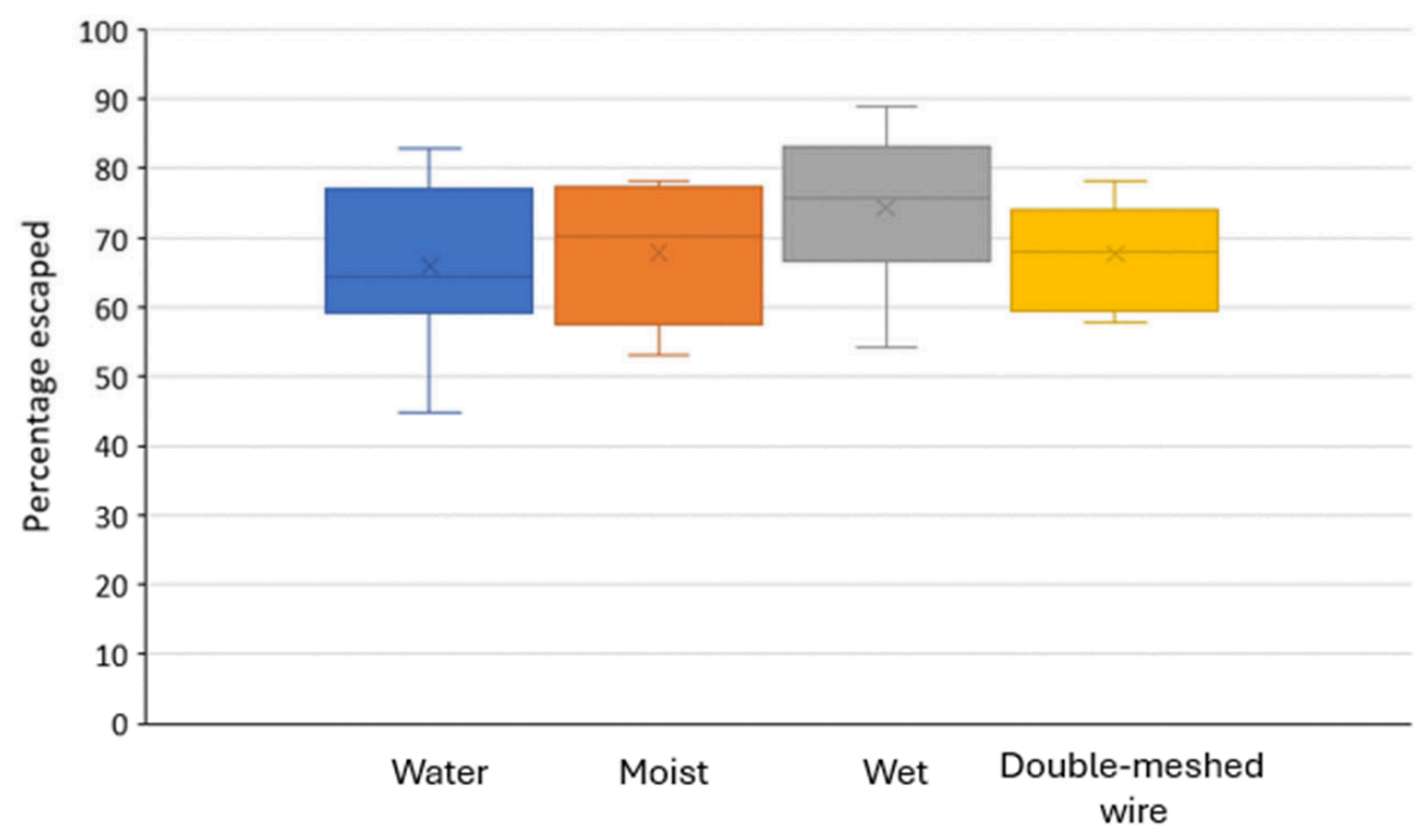
Percentage of escaped male *Ae. aegypti* mosquitoes that emerged from the four substrates. The middle line in the box indicates the median, while the cross indicates the mean value. The bottom of the box indicates the 1st quartile while the top of the box indicates the 3rd quartile. The ends of the whiskers extending from the box represent the minimum and maximum values that are not outlier values.

Male *Ae. aegypti* mosquitoes from all groups demonstrated similar flight ability, as reflected by the escape rate in the Flight Test Device (Figure 3). The Kruskal–Wallis test indicated no significant difference in escape rates of male mosquitoes across the groups, H(3) = 2.343, *p* = 0.504.

### 3.3. Impact of Substrates on Longevity of Ae. aegypti Mosquitoes

**Figure 4:**
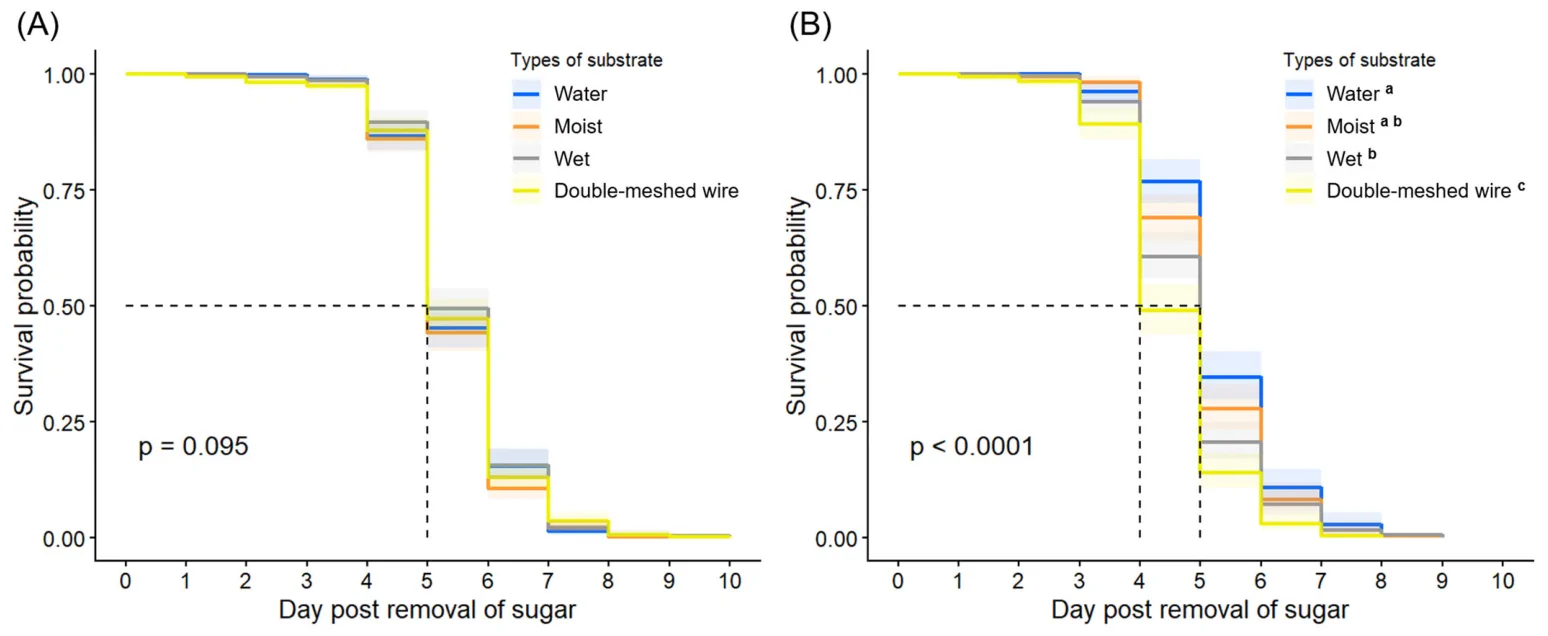
Kaplan–Meier survival curves of (**A**) male and (**B**) female *Ae. aegypti* mosquitoes that emerged from the four substrates. The *p*-value in each chart is the overall *p*-value of the log-rank test. The dashed lines indicate the median survival probability of the mosquitoes emerging from each substrate and their corresponding median survival times. Different superscript letters indicate significant differences between groups based on pair-wise log-rank comparisons with Bonferroni correction (statistical significance at *p* < 0.008).

The aggregated survival data indicated that the median (0.5 survival probability) survival time for all male mosquitoes was five days after the sugar removal, irrespective of the substrates they had emerged from (Figure 4). On the other hand, Kaplan–Meier survival analysis showed a significant difference between the survival curves of the female mosquitoes according to substrate type, log-rank χ^2^(3) = 71.5, *p* < 0.0001. The median survival time for all female mosquitoes emerging from water, moist and wet substrates occurred between four and five days after sugar removal. Female mosquitoes emerging from double-meshed wire showed slightly shorter median survival at day 4. Female mosquito survival was significantly lower following emergence from double-meshed wire compared to other substrates. The survival time of females emerging from the wet substrate also differed significantly from those emerging from water (*p* < 0.008). The survival curves for the respective experimental repeats can be found in Figures S2–S6.

### 3.4. Impact of Emergence Substrates on Mating Success of Ae. aegypti Mosquitoes

**Figure 5:**
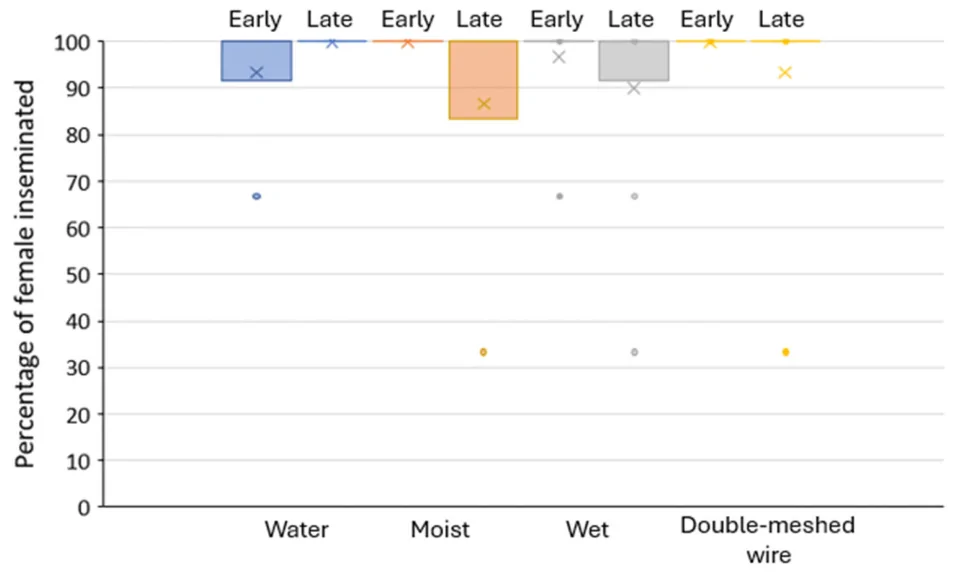
Mating propensity of male *Ae. aegypti* mosquitoes that emerged from four substrates towards female *Ae. aegypti* mosquitoes that emerged from water and were 1 day younger than the males. “Early” refers to mosquitoes that emerged from pupae within 19 h after being placed on the substrate; “Late” refers to mosquitoes that emerged after 28 h after being placed on the substrate. A cross represents the mean value while a dot represents an outlier. The columns indicate the interquartile range.

The substrate from which the male *Ae. aegypti* mosquitoes emerged had little impact on the ability of the males to inseminate female *Ae. aegypti* mosquitoes. This observation holds for both groups of male mosquitoes—those that emerged from pupae within 19 h (Early) after being placed on the substrate, as well as those that emerged after 28 h (Late) (Figure 5).

Similarly, with the exception of two females that emerged from the moist substrate and double-meshed wire, all female *Ae. aegypti* mosquitoes mated successfully regardless of the duration that female *Ae. aegypti* pupae spent on the substrate prior to emergence.

Overall, insemination success was high across all substrate conditions in both male and female mating assays. In the male assay, a significant Age × Substrate interaction was detected (χ^2^ = 9.326, df = 3, *p* = 0.025), indicating that the pattern of insemination success across substrates differed between early and late males. Early males maintained high insemination success across all substrates, with no significant substrate effect (*p* = 0.196). Late males also showed high insemination success, although there was a weak non-significant trend towards reduced success under some substrates (*p* = 0.099). In the female assay, there was no significant Age × Substrate interaction (*p* = 1.000), no significant substrate effect (*p* = 0.392), and no significant age effect (*p* = 0.084). These results indicate that mating/insemination ability was generally retained across substrates, although late males showed some evidence of greater substrate-related variation.

### 3.5. Impact of Substrates on the Emergence of Ae. albopictus, Cx. quinquefasciatus and An. epiroticus Mosquitoes

**Figure 6:**
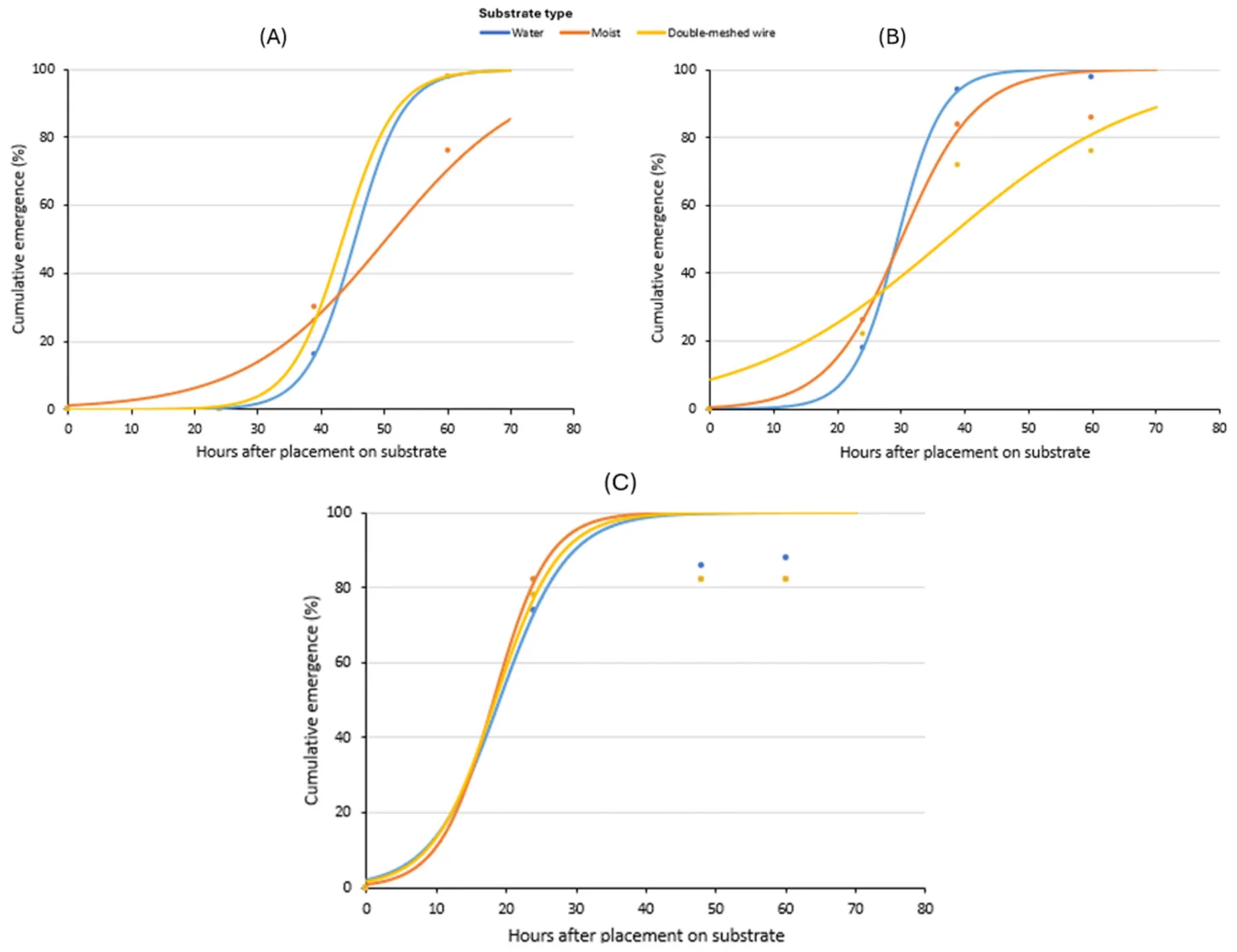
Emergence rates of mosquito species on different substrates: (**A**) *Ae. albopictus* mosquitoes; (**B**) *Cx. quinquefasciatus* mosquitoes; and (**C**) *An. epiroticus* mosquitoes. The dots indicate the data points.

The emergence percentage of male and female *Ae. albopictus*, *Cx. quinquefasciatus* and *An. epiroticus* mosquitoes on moist paper and meshed wire ranged from 76% to 98%, compared to 88% to 98% in water (Table 2). Visual inspection of the graphs revealed no apparent differences in emergence rates across substrates within each species (Figure 6).

## 4. Discussion

The current study demonstrates that mosquito pupae can successfully emerge when laid on their side on any moist substrate and not necessarily upright in water (refer to the Supplementary Video S1). The emergence of *Aedes aegypti* adults from pupae left on moist blotting paper has been previously documented [2], with *Ae. aegypti* pupae that were kept moist on filter paper emerging into adults within four days [3]. A separate report also showed that pupae which were 24- to 48 h old emerged into adults within 6 h after removing them from water [4]. The hardened pupal cuticle is considered highly resistant to loss of water due to desiccation, while larvae quickly perish without moisture [2]. Similarly, pupae survived on the moist setup (Figure 1b), which was not provided with continuous moisture in this study. The filter paper dried eventually, yet the remaining pupae survived. When dispensed properly and with adequate moisture, more than 94% of viable *Ae. aegypti* pupae survived and emerged into adults on all the tested substrates.

The *Ae. aegypti* adults had similar longevity, flight and mating success after emerging from pupae placed on the moist surfaces for over 24 h, when compared to those that emerged on water. The statistically significant differences detected in the study were as follows: (i) the percentages of male and female mosquitoes that successfully emerged on the different substrates; (ii) the longevity of female *Ae. aegypti* that emerged on different substrates; and (iii) Age × Substrate interaction indicating that the pattern of insemination success differed between early and late males, with late males showing greater substrate-related variation. However, substrate effects were not statistically significant when early and late males were analysed separately, so this interaction should be interpreted cautiously. In females, insemination success was near complete across treatments, and no significant substrate or age effects were detected. Given the modest sample size and low number of non-inseminated individuals, these findings indicate no detectable reduction in mating ability under the tested conditions.

The broader operational implications of these findings are considerable. Transporting mosquito pupae on moist substrates can significantly enhance the release logistics of mosquito control programmes by eliminating the need for large volumes of water, thereby reducing transport costs and logistical complexity. Additionally, reduced water volume minimises pupal movement during transport, decreasing the risk of physical damage to pupae and improving overall quality. These advantages, combined with a lowered risk of spillage, and easier handling and transfer, are particularly advantageous for long-distance or transboundary shipments [6,9,10,11]. Adult male mosquitoes require immobilisation, specialised packaging and long-distance chilling, which may potentially impact their fitness [12,13], whereas pupae could be transported unchilled or at about 15–18 °C (as demonstrated for *Ae. albopictus*) [12]. Furthermore, emergence timing could be regulated to coincide with arrival, preventing premature emergence during transport, mitigating the risk of accidental release and reducing the need for stringent temperature control during transport.

Whilst established protocols exist for transporting pupae in water, standardised guidelines for water-free pupal transport remain to be developed. It is important to note that whilst the present study demonstrates that moist substrate emergence does not compromise adult fitness under laboratory conditions, the additional stresses of long-distance physical transportation represent distinct challenges that warrant further investigation. Previous studies have highlighted that packing density and life stage are critical factors in non-chilled transport of sterile male mosquitoes, with overcrowding shown to negatively affect survival, longevity, and mating competitiveness [14]. Notably, transporting pupae under non-chilled conditions in a small volume of water was found to be feasible [14,15], though restricted by overcrowding effects at higher packing densities [14]. Several research questions therefore remain unresolved in the context of long-distance water-free pupal transport, including optimal packing densities, temperature ranges across species, and the potential impact of transport conditions on post-emergence mating competitiveness in the field. Addressing these gaps and establishing standardised packaging and transport guidelines will be critical to the broader implementation of water-free pupal transport.

By transporting pupae, release programmes can also consider pupae releases [15] via water-free pupae release stations, without the risk of mosquito breeding. Doing so would allow for flexibility in the choice of adult mosquito or pupae release methods in Sterile Insect Technique (SIT) and Insect Incompatible Technique (IIT) programmes, tailored to the local context. However, this deployment method needs to be validated across different field contexts and geographical settings, and providing a readily available sugar source for the newly emerged adults can be a challenge [16].

**Figure 7:**
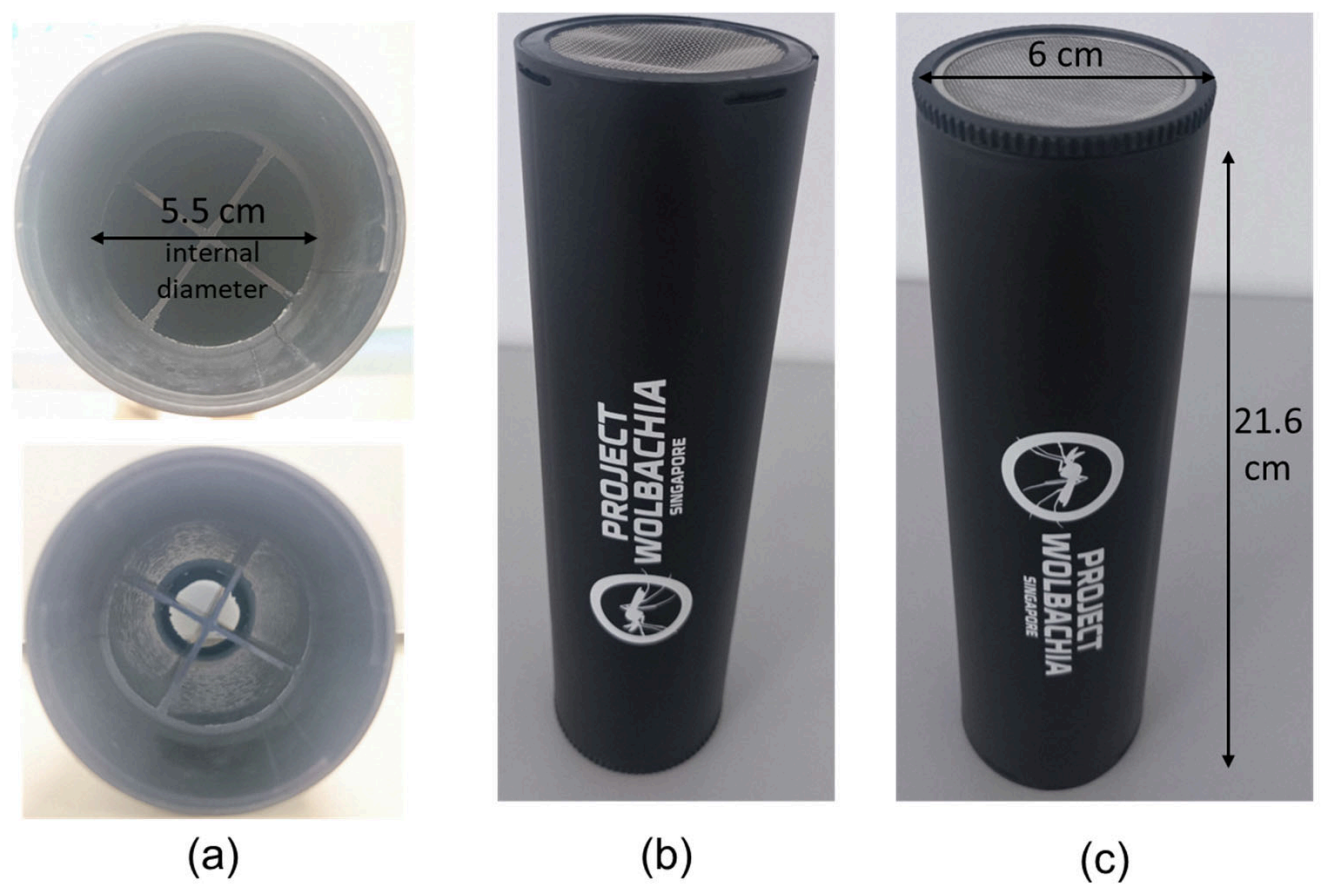
Design features of the Mosquito Launcher. (**a**) Interior view of the Mosquito Launcher, with a cross-sectional design element for an additional resting surface for the mosquitoes; (**b**) exterior view of the Mosquito Launcher; (**c**) an inverted Mosquito Launcher, showing the double-meshed cap at the top.

The practical benefits from these findings are demonstrated by Project *Wolbachia*-Singapore’s use of the Mosquito Launchers (Orinno Technology Pte. Ltd., Singapore) (Figure 7) in its release programme. Known quantities of male pupae are dispensed onto double-meshed caps (Figure 1d) without overcrowding, allowing them to emerge without water submersion and to be contained within the Mosquito Launcher with an internal cross-sectional design element that offers an additional resting surface. The mosquitoes are provided with a sugar solution via soaked cloth strips placed on top of the Mosquito Launcher. This offers a practical advantage over the All-in-One mosquito container that requires the containers to be left submerged in water [17]. The male adult mosquitoes are then released as early as day 3 after placement in the Mosquito Launcher, by simply uncapping the launchers. This innovative approach has demonstrably led to space savings and increased efficiency [18] of mass production and release, facilitating the project’s expansion.

While SIT and IIT programmes primarily transport male mosquitoes, female longevity remains relevant in other vector research and control approaches, such as the *Wolbachia* replacement strategy, which requires the release of both sexes. The reason for the observed sex-specific differences in female longevity across substrates remains unknown, and may reflect underlying differences in pupal cuticle biology between sexes, including cuticle composition, body water content, and desiccation resistance. Future investigations should control for pupal age at collection, given the known developmental rate differences between sexes. Additionally, the fitness parameters of other mosquito species were not assessed in this study and should be explored in future research, particularly in the context of mass rearing and transport for vector control programmes.

Beyond its implications for mosquito release programmes, the ability of pupae to survive on moist substrates after displacement from their aquatic habitats due to erratic weather events, such as (flash) floods, is noteworthy from a public health perspective. These pupae may remain viable on moist grounds and subsequently emerge as adults, potentially necessitating the development of alternative mosquito surveillance strategies to detect these cryptic sites of emergence. Similarly, there is a risk associated with the disposal of breeding receptacles containing immature mosquitoes without proper treatment. Simply discarding or draining water from breeding containers and leaving them in place creates ongoing risk, as viable pupae may survive on residual moisture and subsequently emerge as adults. Effective source reduction therefore requires comprehensive actions that go beyond mere water removal: potential breeding habitats must be permanently removed, modified to prevent water accumulation, securely covered, or physically destroyed through methods such as overturning containers or breaking them.

Overall, these findings open avenues for further investigations and R&D efforts to cater for various mosquito-related needs and for developing robust field and operation protocols.

## Figures and Tables

**Table 1:** Percentages of *Ae. aegypti* mosquitoes that successfully emerged from different substrates.

	Percentage of Pupae That Emerged			
	Water	Moist	Wet	Double-Meshed Wire
Male *Ae. aegypti* *p* value against water	99.63 ^a,b^ (*n* = 1347, S.D. = 0.49)	94.64 ^a^ (*n* = 1343, S.D. = 3.82) 0.0002	97.85 (*n* = 1349, S.D. = 2.44) 0.2082	96.89 ^b^ (*n* = 1349, S.D. = 2.08) 0.0101
Female *Ae. aegypti* *p* value against water	99.56 ^c,d,e^ (*n* = 1347, S.D. = 0.58)	95.04 ^c^ (*n* = 1349, S.D. = 3.25) 0.0002	97.11 ^d^ (*n* = 1348, S.D. = 2.23) 0.0208	96.15 ^e^ (*n* = 1350, S.D. = 2.30) 0.0016

^a–e^ Dunn’s test post hoc analysis showing significant differences (adjusted *p* < 0.05) between the pair indicated by the same letters.

**Table 2:** Percentages of mosquitoes that successfully emerged from different substrates.

	Water	Moist	Double-Meshed Wire
*Ae. albopictus* (*n* = 50)	98%	76%	98%
*Cx. quinquefasciatus* (*n* = 50)	98%	86%	76%
*An. epiroticus* (*n* = 50)	88%	82%	82%

## Data Availability

Data are contained within the article or Supplementary Material.

## Supplementary Materials

The following supporting information can be downloaded at: https://www.mdpi.com/article/10.3390/insects17080770/s1. Figure S1: Surface temperatures of the substrates at different timepoints after sampling. The error bars denote standard deviations. Figure S2: Kaplan–Meier survival curve of male *Ae. aegypti* mosquitoes from the first longevity test. Figure S3: Kaplan–Meier survival curve of male *Ae. aegypti* mosquitoes from the second longevity test. Figure S4: Kaplan–Meier survival curve of male *Ae. aegypti* mosquitoes from the third longevity test. Figure S5: Kaplan–Meier survival curve of female *Ae. aegypti* mosquitoes from the first longevity test. Figure S6: Kaplan–Meier survival curve of female *Ae. aegypti* mosquitoes from the second longevity test. Supplementary Video S1: Emergence of adult mosquitoes from pupae on wet surfaces.
