# Supplementary material for: “Water? Which Aedes aegypti Pupa Needs It!”
Source: Insects. 2026 Jul 27;17(8):770. doi: 10.3390/insects17080770 (PMC13513539; doi:10.3390/insects17080770)
Supplement: Supplementary file 1 [file insects-17-00770-s001.zip › Supplementary Materials.pdf]

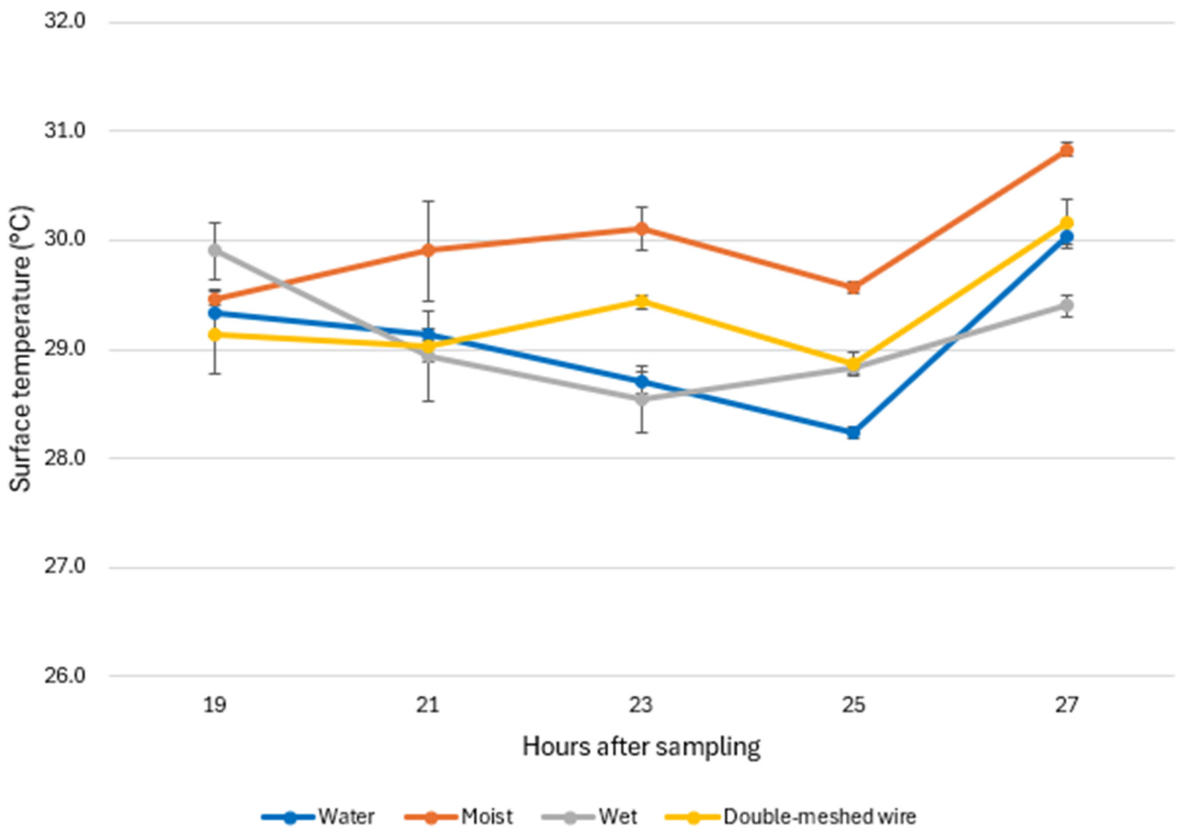

Figure S1. Surface temperatures of the substrates at different timepoints after sampling. The error bars denote standard deviations.

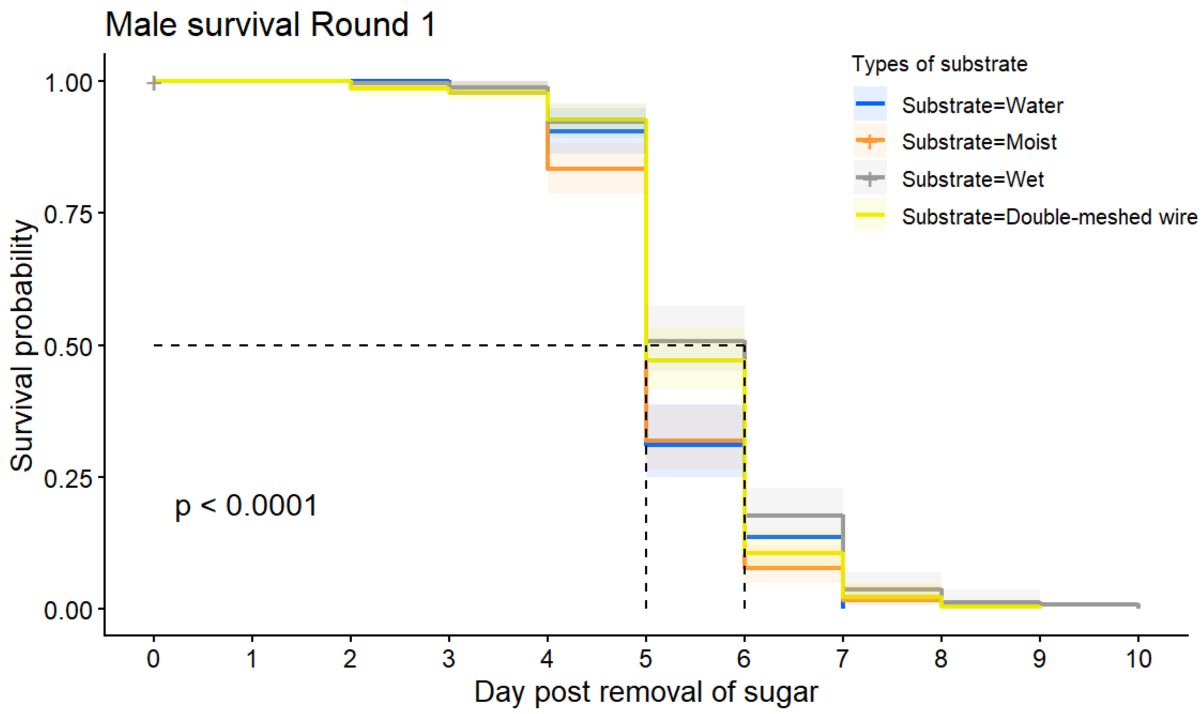

Figure S2. Kaplan-Meier survival curve of male *Ae. aegypti* mosquitoes from the first longevity test.

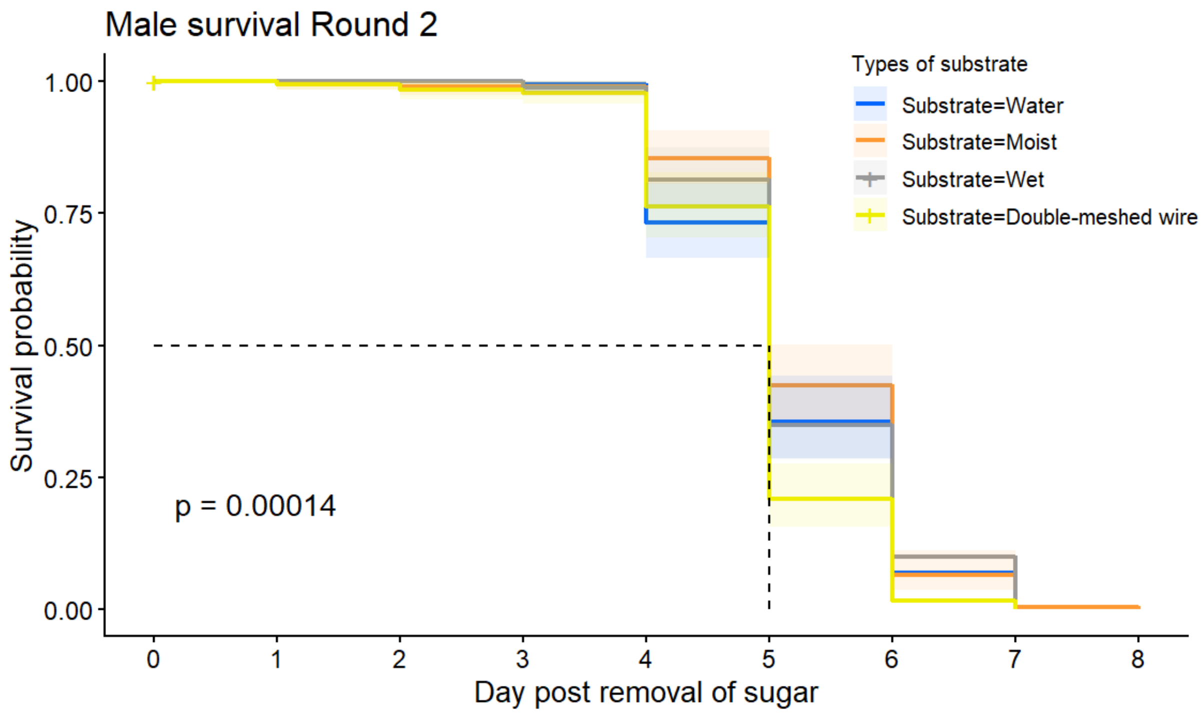

Figure S3. Kaplan-Meier survival curve of male *Ae. aegypti* mosquitoes from the second longevity test.

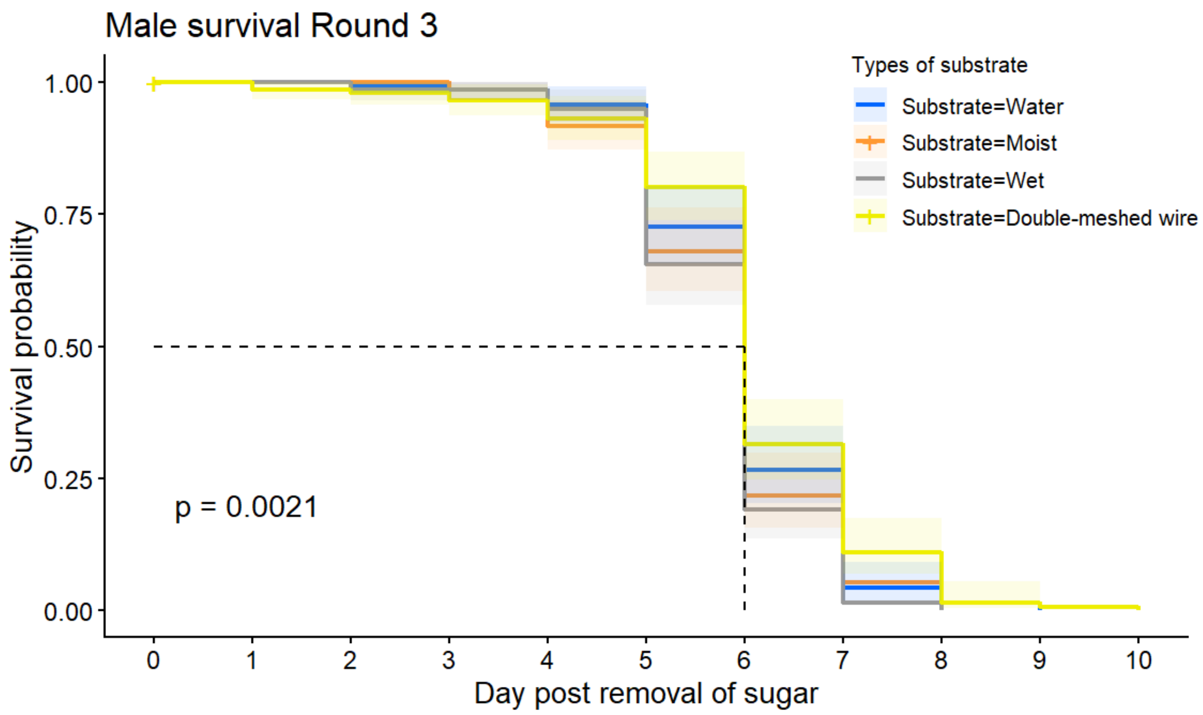

Figure S4. Kaplan-Meier survival curve of male *Ae. aegypti* mosquitoes from the third longevity test.

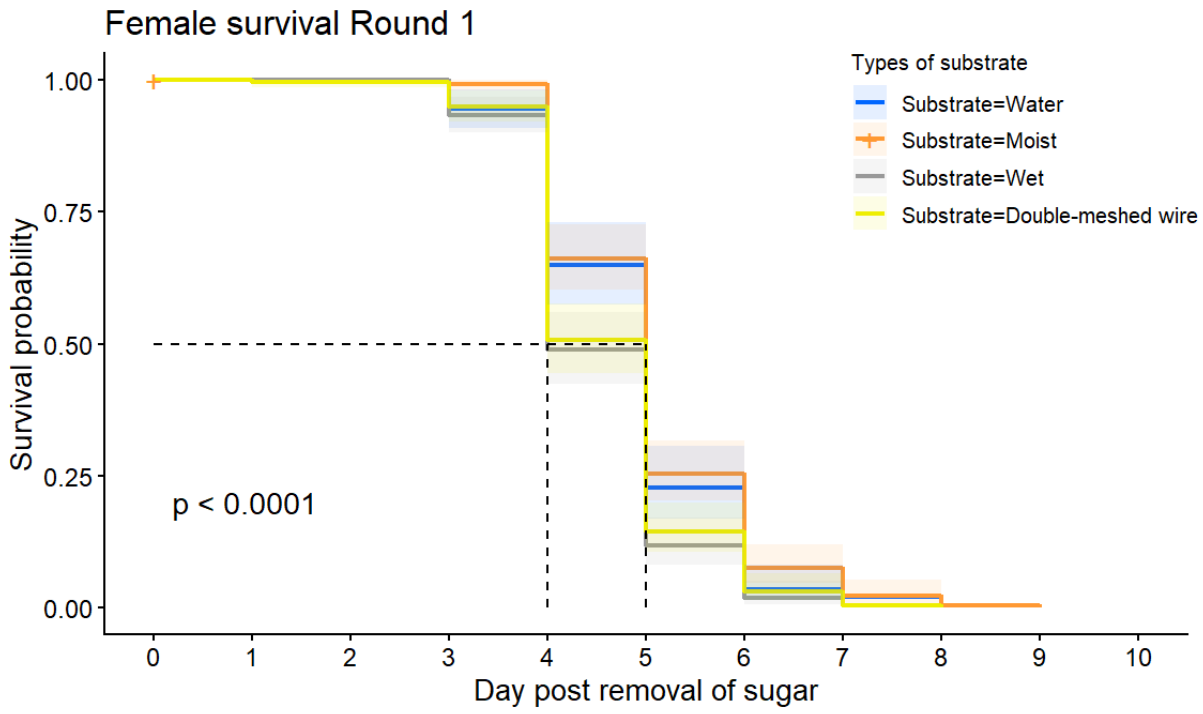

Figure S5. Kaplan-Meier survival curve of female *Ae. aegypti* mosquitoes from the first longevity test.

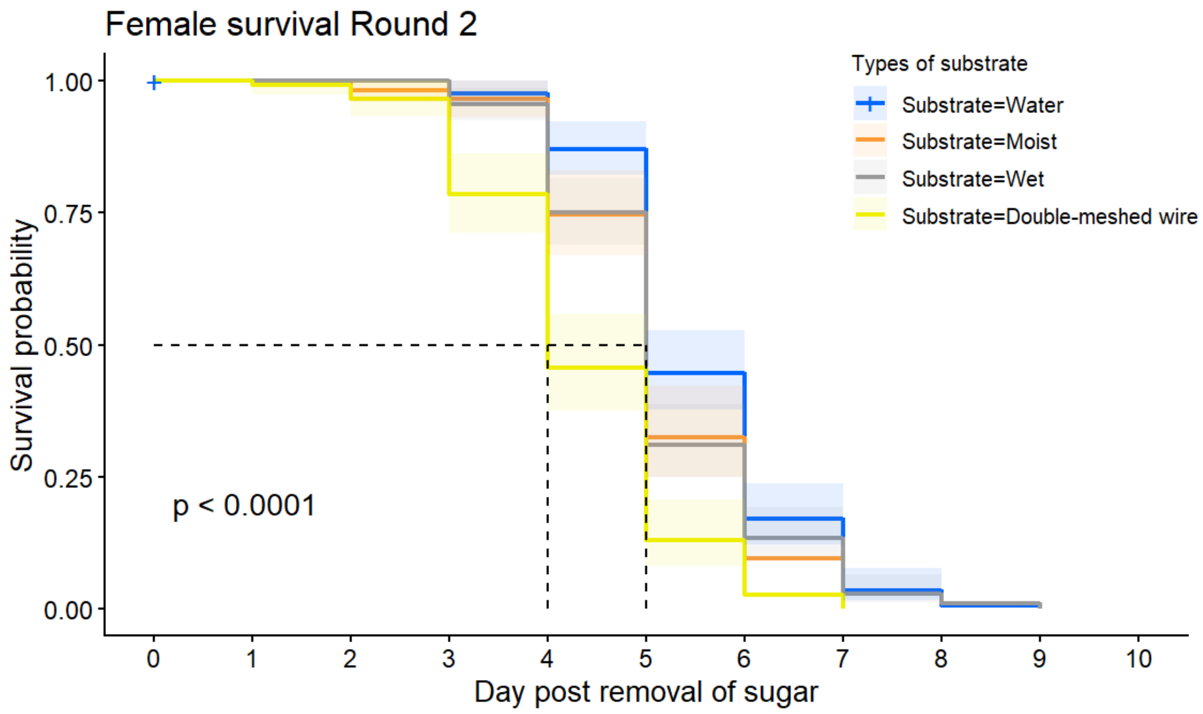

Figure S6. Kaplan-Meier survival curve of female *Ae. aegypti* mosquitoes from the second longevity test.
